# Characterization of Oxacillin-Resistant and Oxacillin-Susceptible *mecA*-Positive *Staphylococcus pseudintermedius* from Skin Lesions and Nasal Cavities of Dogs with Clinical Pyoderma

**DOI:** 10.3390/ani14172613

**Published:** 2024-09-08

**Authors:** Putu Ayu Sisyawati Putriningsih, Jaruwan Kampa, Suphattra Jittimanee, Patchara Phuektes

**Affiliations:** 1 Graduate School, Faculty of Veterinary Medicine, Khon Kaen University, Khon Kaen 40002, Thailand; putu_ayu_sisyawati@kkumail.com; 2 Laboratory of Veterinary Internal Medicine, Faculty of Veterinary Medicine, Udayana University, Denpasar 80361, Indonesia; 3 Division of Pathobiology, Faculty of Veterinary Medicine, Khon Kaen University, Khon Kaen 40002, Thailand; jarpat@kku.ac.th (J.K.); suphattra@kku.ac.th (S.J.)

**Keywords:** *Staphylococcus pseudintermedius*, *mecA*, dogs, pyoderma, oxacillin-resistant, oxacillin-susceptible

## Abstract

**Simple Summary:**

*Staphylococcus pseudintermedius* is a common bacterium that causes skin infections in dogs and can occasionally infect humans, posing a public health risk. The presence of the *mecA* gene in this bacterium can lead to resistance against methicillin (oxacillin) and other beta-lactam antibiotics, which limits treatment options. This study investigated the characteristics of oxacillin-resistant and oxacillin-susceptible *mecA*-positive *S. pseudintermedius* isolates from the skin and nasal cavities of dogs with pyoderma. We found a high prevalence of multidrug resistance and significant genetic diversity among the isolates, both within the dog itself and among others, highlighting the complexity of *S. pseudintermedius* colonization and infection dynamics in pyoderma dogs. Careful monitoring and treatment strategies are essential to manage the spread of antibiotic resistance effectively.

**Abstract:**

Understanding the epidemiology of *mecA*-positive *Staphylococcus pseudintermedius* strains, including those that are oxacillin-susceptible but potentially inducible to resistance, is crucial for developing effective treatment strategies and mitigating public health risks. This study characterized 87 *mecA*-positive *S. pseudintermedius* isolates obtained from skin lesions and nasal orifices of 46 dogs with pyoderma enrolled at a referral hospital in Thailand between 2019 and 2020. All isolates underwent antibiogram profiling, SCC*mec* typing, and pulsed-field gel electrophoresis (PFGE) for phenotypic and genetic analysis. Among the 87 isolates, 33 isolates (37.9%) recovered from 15 dogs were oxacillin-resistant (OR-MRSP), while 54 isolates (62.1%) from 31 dogs were oxacillin-susceptible (OS-MRSP). All OR-MRSP isolates exhibited multidrug resistance (MDR), and 44% of the OS-MRSP isolates also showed MDR. SCC*mec* typing revealed type V as predominant among OR-MRSP isolates (69.7%), while many oxacillin-susceptible isolates (70.4%) were non-typeable. The OR-MRSP isolates from the same dog showed consistent antibiogram and SCC*mec* types, while OS-MRSP isolates displayed both identical and diverse patterns. No dominant pulsotypes were observed among the OR-MRSP or OS-MRSP strains. Genetic diversity was also noted among the isolates within the same dogs and among the others, highlighting the complexity of *S. pseudintermedius* colonization and infection dynamics in pyoderma-affected dogs.

## 1. Introduction

*Staphylococcus pseudintermedius* is an opportunistic pathogen naturally found on dogs’ skin, mucosae, and other body sites. With abnormalities of the skin condition, the bacteria can also provoke infections of the skin, ears, and other body tissues and cavities [1,2]. *S. pseudintermedius* is a major pathogenic microorganism encountered in companion animal dermatology practice [3] and is mainly found as secondary infections in dogs [4]. Human colonization and, occasionally, human infections by *S. pseudintermedius* have been reported sporadically [3,4,5,6], indicating the possibility that this bacterium can be zoonotic and may pose a public health problem.

Methicillin-resistant *S. pseudintermedius* (MRSP) is a strain of *S. pseudintermedius* that exhibits resistance to methicillin and penicillinase-stable beta-lactam antibiotics. This resistance is conferred by the *mecA* gene, which encodes a modified penicillin-binding protein 2a (PBP2a) [1]. MRSP is considered a reservoir for resistant genes to various antibiotics [7], as MRSP isolates are frequently multidrug-resistant (MDR), which is defined as resistance to at least one agent in three or more classes of antimicrobials [8]. In recent years, the frequency of MRSP detection in microbiological samples from dogs and cats has increased [1], posing a significant threat to veterinary medicine due to the limited therapeutic options available [9].

Apart from methicillin resistance, methicillin (oxacillin)-susceptible *mecA*-positive staphylococci have been increasingly identified globally [10,11,12,13,14]. Reports have documented *Staphylococcus aureus* isolates carrying the *mecA* gene while remaining phenotypically susceptible to oxacillin, termed oxacillin-susceptible MRSA (OS-MRSA). OS-MRSA can be inducible to resistance after antibiotic exposure [15], resulting in therapeutic failures with beta-lactam antimicrobials [16]. Importantly, a recent study detected OS-MRSA isolates in the nasal mucosa of healthy dogs and their owners [17]. In the case of *S. pseudintermedius*, the presence of *mecA*-positive strains that remain oxacillin-susceptible has not been comprehensively investigated and characterized. Given that *mecA* PCR is not routinely employed in laboratories, these strains may be unobserved, potentially facilitating their silent dissemination and posing challenges for effective treatment in infected dogs, particularly with bata-lactam antibiotics. Our previous study demonstrated a high prevalence of *mecA*-positive *S. pseudintermedius* in skin lesions of dogs with pyoderma; however, these strains have yet to be thoroughly characterized [18]. Therefore, this study aims to characterize and compare oxacillin-resistant and oxacillin-susceptible *mecA*-positive *S. pseudintermedius* isolates from dogs with clinical pyoderma. We also aim to compare infecting and colonizing strains within individuals and among the studied group. Understanding the epidemiology and characteristics of *mecA*-positive *S. pseudintermedius* strains is crucial for developing effective treatment strategies in veterinary clinical settings and for mitigating potential public health risks. 

## 2. Materials and Methods

### 2.1. Sample Collection 

Eighty-seven *S. pseudintermedius* isolates included in this study were obtained from 46 out of 63 enrolled dogs with pyoderma that visited the Veterinary Teaching Hospital, Faculty of Veterinary Medicine, Khon Kaen University, between September 2019 and September 2020. Two skin swabs were collected from two separate lesions on each dog, and one nasal swab was obtained from each enrolled dog. These swabs were placed in a transport medium and stored at 4 °C before being cultured on blood agar, which contains a blood agar base supplemented with 5% defibrinated sheep blood (Clinical Diagnostics, Bangkok, Thailand). Inoculated plates were subsequently incubated at 37 °C under aerobic conditions for 18 to 48 h. 

### 2.2. Isolation and Identification of Oxacillin-Resistant and Oxacillin-Susceptible mecA-Positive Staphylococcus pseudintermedius

Staphylococci-like colonies grown on sheep blood agar were subjected to species identification based on their phenotype and genotype. All 87 isolates were phenotypically identified as *S. pseudintermedius* using the VITEK^®^ 2 system (Biomerieux, Marcy l’etoile, France) and genotypically confirmed through polymerase chain reaction (PCR) targeting the *nuc* gene [19]. The *mecA* gene in each *S. pseudintermedius* isolate was detected using conventional PCR, following the protocol developed by Olivera [20], as previously described [18]. 

Antimicrobial susceptibility testing was performed using the VITEK^®^ 2 system to identify oxacillin-resistant and oxacillin-susceptible isolates (Biomerieux, France). The antibiotics tested included oxacillin (OXA), benzylpenicillin (BEN), amoxicillin/clavulanic acid (AMC), cephalothin (CEP), cefpodoxime (CPD), cefovecin (CEV), amikacin (AMK), gentamicin (GEN), enrofloxacin (ENR), marbofloxacin (MAR), pradofloxacin (PRA), erythromycin (ERY), clindamycin (CLI), doxycycline (DOX), minocycline (MIN), nitrofurantoin (NIT), chloramphenicol (CHL), florfenicol (FLO), and trimethoprim/sulfamethoxazole (SXT). The minimum inhibitory concentration (MIC) of oxacillin was set at ≥0.5 μg/mL as an indicator of methicillin resistance; resistance to other antimicrobial agents was determined according to the recommendations of the Clinical and Laboratory Standards Institute (CLSI) [21]. A disk diffusion test was also performed using a 1 µg oxacillin disk, with a cut-off of <20 mm used to determine resistance or susceptibility [22].

### 2.3. Pulsed-Field Gel Electrophoresis (PFGE)

The pulsed-field gel electrophoresis (PFGE) protocol was based on a prior study [23] with minor modifications. A CHEF-DR III system (Bio-Rad Laboratories, CA, USA) was used to conduct PFGE. All genomic DNA plugs were initially digested with *Sma*I (New England BioLabs Inc., MA, USA). Those isolates not digested by *Sma*I were subsequently digested with *Xma*I (New England BioLabs Inc., MA, USA). Cluster analysis was performed using the Unweighted Pair Group Method with Arithmetic Mean (UPGMA) in BIO-PROFIL Bio-1D++ software (Vilber Lourmat, Eberhardzell, Germany), employing the Dice similarity coefficient with a 2% interval of confidence for band matching. Isolates were clustered based on an 80% similarity cut-off.

### 2.4. SCCmec Typing

SCC*mec* typing was performed using two multiplex PCR sets (M-PCR 1 and M-PCR 2) described by Kondo et al. [24]. The first set (M-PCR 1) was used to detect the *mecA* gene and identify the *ccr* gene complex, while the second set (M-PCR 2) was employed to identify the *mec* gene complex. For M-PCR 1, the reaction mixture in a total volume of 25 µL contained 1x Qiagen Multiplex PCR Master Mix (Qiagen, Tegelen, The Netherlands), 10 pmol of each primer, 2 µL of extracted DNA, and RNase-free water to make up the volume. The PCR cycles consisted of an initial denaturation at 95 °C for 15 min, followed by 35 cycles of 94 °C for 2 min, 57 °C for 90 s, 72 °C for 2 min, and a final extension at 72 °C for 10 min. For M-PCR 2, the reaction mixture and reaction conditions were the same as for M-PCR 1, except that the annealing temperature was changed to 60 °C. DNA fragments were analyzed by electrophoresis in 1x TBE buffer on a 1.5% agarose gel stained with 1x Redsafe™ (iNtRON Biotechnology Inc., Seongnam, Republic of Korea).

### 2.5. Statistical Analysis

Statistical analyses were performed to assess the prevalence and associations of the isolates. Prevalence was calculated as a proportion of the total number of isolates or dogs and corresponding confidence intervals (CIs). The two-tailed Fisher’s exact test was used to evaluate the association between genotypic characteristics and phenotypes. This test was chosen due to the categorical nature of the data and the small sample sizes in some categories. A *p*-value of less than 0.05 was considered statistically significant. All statistical analyses and heatmaps were performed using GraphPad Prism version 10.0 for Windows (GraphPad Software, Boston, MA, USA; www.graphpad.com (accessed on 3 August 2024).

## 3. Results

### 3.1. Sample Origins and Distribution of S. pseudintermedius Isolates Positive for the mecA Gene

The 87 *S. pseudintermedius* isolates positive for the *mecA* gene comprised 67 skin isolates and 20 nasal isolates, which were recovered from 46 dogs out of 63 dogs (73%, 95% CI: 60.3–83.4). Only one isolate was obtained from each sampling site of an individual dog. Single skin isolates were obtained from nine dogs, and single nasal isolates from another six dogs. Seventeen dogs yielded two skin isolates each, while four dogs yielded both a skin and a nasal isolate. Ten dogs yielded two skin isolates and one nasal isolate, as shown in Table 1. 

Based on an MIC value of ≥0.5 μg/mL and a zone diameter of <20 mm for a 1 µg oxacillin disk, 33 out of 87 isolates (37.9%) were identified as OR-MRSP, while 54 isolates (62.1%) were classified as OS-MRSP. The MIC values of the isolates were consistent with the results of the disk diffusion test. Among the 33 OR-MRSP isolates, there were 24 skin isolates and 9 nasal isolates, obtained from 15 of the 63 enrolled dogs, representing 23.8% (95% CI: 14.0–36.2). The 54 oxacillin-susceptible isolates included 43 skin isolates and 11 nasal isolates from 31 dogs, accounting for 49.2% of 63 dogs (95% CI: 36.4–62.1).

### 3.2. Phenotypic Characteristics of Oxacillin-Resistant and Oxacillin-Susceptible mecA-Positive S. pseudintermedius 

**Figure 1:**
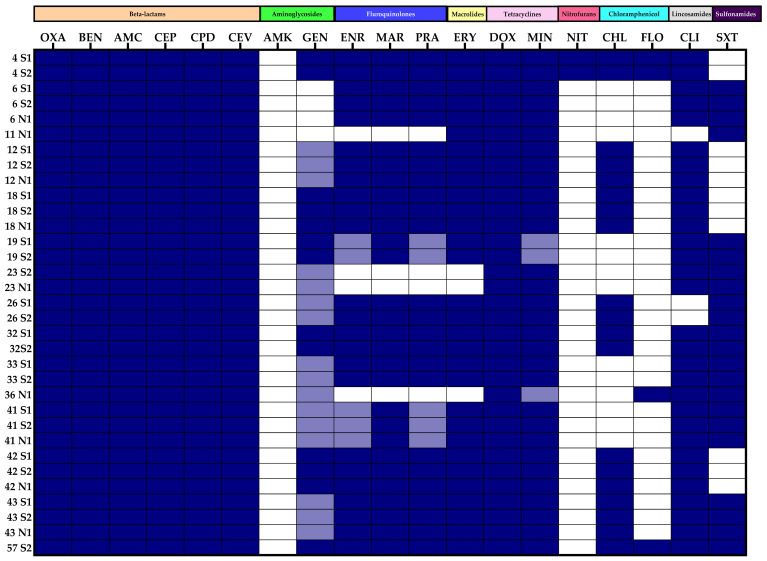
Heat map illustrating the antibiogram patterns of 33 oxacillin-resistant *mecA*-positive *Staphylococcus pseudintermedius* (OR-MRSP) isolates. Antimicrobial susceptibility was assessed using the VITEK^®^ 2 system. Each row represents an individual isolate, labelled by the dog number from which it was obtained, the sample origin (skin lesion or nasal swab), and the corresponding isolate number from each dog. The heat map employs a color gradient where dark blue boxes indicate resistance, light blue boxes represent intermediate susceptibility, and white boxes represent susceptibility to the tested antibiotics.

**Figure 2:**
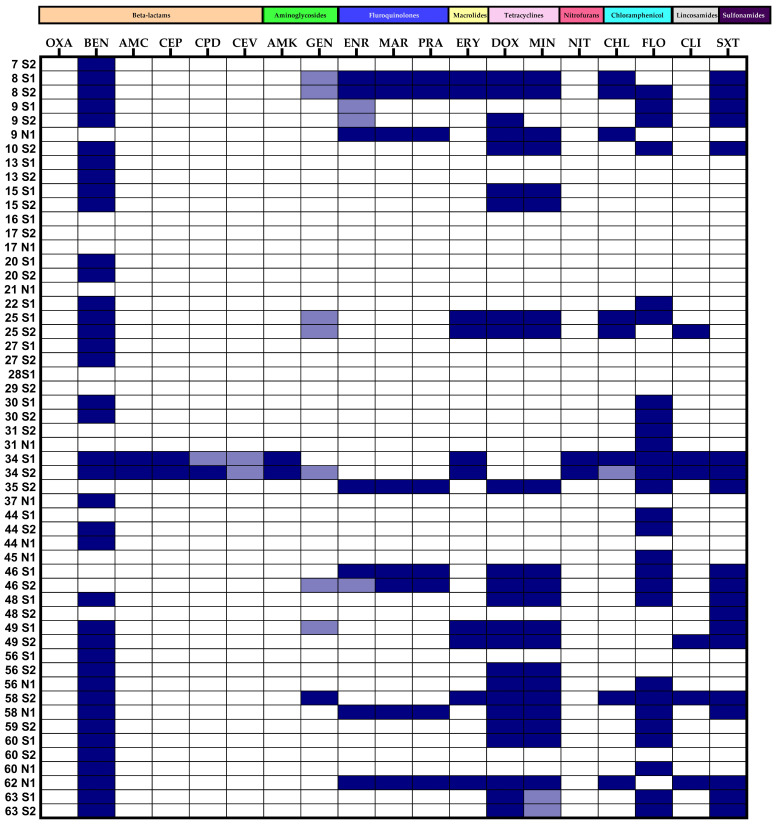
Heat map illustrating the antibiogram patterns of 54 oxacillin-susceptible *mecA*-positive *Staphylococcus pseudintermedius* (OS-MRSP) isolates. Antimicrobial susceptibility was assessed using the VITEK^®^ 2 system. Each row represents an individual isolate, labelled by the dog number from which it was obtained, the sample origin (skin lesion or nasal swab), and the corresponding isolate number from each dog. The heat map employs a color gradient where dark blue boxes indicate resistance, light blue boxes represent intermediate susceptibility, and white boxes represent susceptibility to the tested antibiotics.

A total of 40 different antibiogram patterns were identified among the 87 *S. pseudintermedius* isolates, with no predominant antibiogram observed (Figure 1 and Figure 2). All OR-MRSP isolates exhibited multidrug resistance (MDR). Of 33 oxacillin-resistant isolates, 14 antibiogram patterns were identified. The most prevalent MDR pattern among OR-MRSP isolates was resistant to 15 antibiotics including oxacillin, benzylpenicillin, amoxicillin/clavulanic acid, cephalothin, cefpodoxime, cefovecin, gentamicin, enrofloxacin, marbofloxacin, pradofloxacin, erythromycin, clindamycin, doxycycline, minocycline, and chloramphenicol. This pattern was observed in 6 out of the 33 OR-MRSP isolates (18.2%). These six isolates were derived from skin lesions (four isolates) and nasal cavities (two isolates) of two dogs (Figure 1). 

Additionally, 24 out of 54 (44.4%) OS-MRSP isolates, derived from skin lesions (20 isolates) and nasal cavities (4 isolates) of 15 dogs, exhibited multidrug resistance, with a total of 20 distinct MDR patterns observed. However, a significant association was found between OR-MRSP isolates and multidrug resistance (MDR) (*p* ≤ 0.0001), but not with oxacillin-susceptible isolates. Of 26 antibiogram patterns identified among OS-MRSP isolates, the most prevalent pattern was resistance solely to benzylpenicillin (BEN), which was detected in 11 isolates (11/54, 20.4%) originating from skin lesions (9 isolates) and nasal cavities (2 isolates) of eight dogs. Notably, two oxacillin-susceptible isolates from the skin lesions of a single dog exhibited resistance to all beta-lactams tested except for oxacillin (Figure 2).

### 3.3. Genotypic Characteristics of Oxacillin-Resistant and Oxacillin-Susceptible mecA-Positive Staphylococcus pseudintermedius

#### 3.3.1. SCC*mec* Type

Thirty-nine isolates (39/87, 44.8%) harbored the *ccr* gene complex 5 (C) and *mec* gene class C2, classified as SCC*mec* type V. SCC*mec* types could not definitively be assigned to 48 isolates (55.2%) due to their combination of *ccr* and *mec* gene complexes, which could not be classified using the conventional method outlined by the International Working Group on the Classification of Staphylococcal Cassette Chromosome (2009). Among these non-typeable isolates, 46 carried two *ccr* gene complexes, 3 had two *mec* gene complexes, and 9 lacked the *mec* gene complex altogether. One isolate possessed a novel combination of *ccr* and *mec* gene complexes. Details of the SCC*mec* typing results, using two sets of multiplex PCR, are summarized in Table 2.

The majority (23/33, 69.7%) of OR-MRSP isolates were identified as SCC*mec* type V, while the remaining 10 isolates could not be classified (Table 3). In contrast, most oxacillin-susceptible isolates were non-typeable (70.4%), with SCC*mec* type V detected in only 16 isolates (16/54, 29.6%), as shown in Table 4. A significant association was found between SCC*mec* type V and OR-MRSP isolates, as well as between non-typeable SCC*mec* types and OS-MRSP isolates, using Fisher’s exact test (*p* ≤ 0.0004). 

#### 3.3.2. Pulsed-Field Gel Electrophoresis (PFGE) Analysis

Out of a total of 87 MRSP isolates, 65 (74.7%) could be digested by *Sma*I, and 21 (24.1%) by *Xma*I restriction enzymes, while 1 isolate was resistant to digestion by both enzymes. Among the *Sma*I-digested isolates, 49 were from skin and 16 from nasal samples, whereas among the *Xma*I-digested isolates, 18 were from skin and 3 from nasal samples. The undigested isolate originated from a nasal sample. A total of 34 pulsotypes were identified in the *Sma*I-PFGE dendrogram and 14 pulsotypes were identified in the *Xma*I-PFGE dendrogram (Supplementary Figures S1 and S2, Table 3 and Table 4). The PFGE analysis revealed diverse patterns across all 87 isolates, including those from both OR-MRSP and OS-MRSP groups, with no predominant pulsotypes observed. 

### 3.4. Comparative Genotypic and Phenotypic Analysis of S. pseudintermedius Isolates from Individual Dogs and Different Dogs

The genotypic characteristics and multidrug-resistant phenotypes of the 33 OR-MRSP and 54 OS-MRSP isolates from each individual dog are presented in Table 3 and Table 4, respectively. 

Phenotypically, when two or three OR-MRSP isolates were recovered from a single dog, they exhibited the same antibiogram pattern (Table 3, Figure 1). In contrast, OS-MRSP isolates from a single dog were found to exhibit both identical and different patterns, and both multidrug-resistant (MDR) and non-MDR profiles were observed among isolates originating from a single dog (Table 4, Figure 2). 

Genotypically, OR-MRSP isolates from a single dog consistently exhibited the same SCC*mec* type, irrespective of whether they were obtained from skin lesions or nasal cavities, except for two skin isolates from one dog containing a different *ccr* complex (Table 3). The SCC*mec* types of OS-MRSP isolates from the same dog were found to be both uniform and varied, as shown in Table 4. These results parallel the findings of the antibiogram. Based on the chromosomal genomic patterns analyzed by PFGE, no specific patterns were observed in either group. Both OR-MRSP and OS-MRSP isolates from a single dog exhibited both similar and dissimilar pulsotypes. However, isolates with identical pulsotypes were generally derived from different sites on the same dog. Identical pulsotypes were also observed between skin and nasal isolates, while different pulsotypes were noted among skin isolates from individual dogs. With various phenotypes and genotypes observed, no common strains circulated among the dog populations in this study.

## 4. Discussion

The identification and characterization of *mecA*-positive *S. pseudintermedius* isolates from pyoderma-affected dogs provide comprehensive insights into the epidemiology and potential treatment challenges associated with this pathogen and its public health risk. Our study recovered 87 *mecA*-positive *S. pseudintermedius* isolates from 46 out of 63 enrolled dogs (73%). This highlights a notable estimated prevalence of *S. pseudintermedius* carrying the *mecA* gene among dogs affected by pyoderma. The detection of *mecA*-positive isolates in both skin and nasal samples aligns with previous findings [25,26], emphasizing the potential for bacterial colonization in multiple anatomical locations. Notably, nasal isolates of OR-MRSP exhibited identical antibiograms and SCC*mec* types to the infected skin isolates within a single dog. This ability to colonize various sites contributes to its persistent dissemination and potential transfer of resistant genes, thereby complicating treatment and control strategies.

The estimated prevalence of *mecA*-positive *S. pseudintermedius* in our study is concerning, as this gene confers resistance to methicillin and other beta-lactam antibiotics commonly used in veterinary practice [27,28]. The OR-MRSP isolates are generally multidrug-resistant, limiting antibiotic treatment options. Although the impact of OS-MRSP is not well understood, there have been reports indicating that OS-MRSA can become inducibly resistant to oxacillin and other beta-lactams after antibiotic exposure [15]. The mechanisms behind the oxacillin susceptibility of these isolates have been reported to include the absence of a complete SCC*mec* structure except for the *mecA* gene, as well as mutations or insertions in the *mecA* gene or its promoter. Revertant isolates containing mutated genes, different from the parental strains, can revert to a resistant phenotype after cefoxitin exposure [15]. Therefore, the isolates with the *mecA* gene may lead to therapeutic failures, as observed in the previous study involving OS-MRSA in humans [16]. The mechanisms of OS-MRSP isolates in this study necessitate further investigation.

Among the 87 *mecA*-positive *S. pseudintermedius* isolates, 33 (37.9%) were classified as oxacillin-resistant, while 54 (62%) were oxacillin-susceptible. Identifying 40 distinct antibiogram patterns with no predominant pattern suggests significant phenotypic diversity and challenges in selecting effective antimicrobial treatments. Multidrug resistance was observed in 65.5% (57/87) of all *S. pseudintermedius* isolates. In line with previous studies [29,30], our findings, though at a higher proportion, showed that all OR-MRSP isolates exhibited multidrug resistance (MDR), underscoring the significant therapeutic challenges posed by these strains. Additionally, MDR was detected in a significant proportion (24/54, 44.4%) of OS-MRSP isolates. Although most of these isolates were susceptible to extended-spectrum beta-lactam antibiotics, they exhibited resistance to multiple other classes, including tetracyclines, sulphonamides, macrolides, fluoroquinolones, chloramphenicol, and aminoglycosides. A recent study reported an association between the presence of the *mecA* gene and resistance genes to other antibiotic classes, including aminoglycosides, tetracyclines, lincosamides, and macrolides [31]. Interestingly, two oxacillin-susceptible isolates demonstrated resistance to beta-lactam drugs, except oxacillin. The mechanisms underlying this phenotype and other MDR phenotypes of oxacillin-susceptible isolates require further investigation.

SCC*mec* type V was found to be the predominant SCC*mec* type circulating in the study area, located in northeastern Thailand. This finding aligns with global reports indicating that MRSP SCC*mec* types II-III and V are the two main types widely distributed worldwide, including in Europe and North America [7]. Both types are also prevalent in Asia. Most MRSP isolates in North China and Japan have been identified as SCC*mec* type II-III [5,32], while most isolates in South China, Thailand, and Korea have been identified as SCC*mec* type V [33,34,35,36]. In our study, SCC*mec* type V was identified in 69.7% of the OR-MRSP isolates, demonstrating a strong association between this SCC*mec* type and its role in disseminating antimicrobial resistance. In contrast, the majority of OS-MRSP isolates were found to carry non-typeable SCC*mec* elements. The structural differences in SCC*mec* elements could be a potential reason for the large number of non-typeable isolates among OS-MRSP, similar to what has been observed in OS-MRSA [15].

The inability to type a significant portion of isolates using conventional methods suggests the presence of novel or atypical SCC*mec* elements or limitations of the SCC*mec* typing method used. Only two sets of the six multiplex PCRs developed by Kondo et al. [24] were used. According to Turlej et al. [37], these two sets of multiplex PCRs are adequate for characterizing most SCC*mec* elements and are widely used worldwide. These two sets of multiplex PCRs can identify *ccr* gene complexes 1 to 5 and *mec* gene complex classes A, B, and C2. However, *ccr* gene complexes 6 to 9 cannot be detected; thus, SCC*mec* types VII, X, XI, XII, and XIII cannot be identified [38,39]. 

Notably, 38% of the isolates harbored a combination of *ccr* gene complexes 1 and 5 and *mec* gene class C2. This SCC*mec* type was identified in both OR-MRSP and OS-MRSP. A similar result was reported by Chanayat et al. [35], who reported these non-typeable SCC*mec* elements in 33% (4/12) of MRSP isolates from Chiang Mai province, located in northern Thailand. Additionally, our study identified an SCC*mec* combination, comprising *ccr* gene complex class 5 and *mec* gene complex class A in one isolate (1/87, 1%) of OR-MRSP. This specific SCC*mec* combination has also been reported in South Africa [39].

For PFGE analysis, more than one restriction enzyme was needed for genomic DNA digestion in our study. Most of the isolates (74.7%) could be digested with *Sma*I like those reported by previous studies [7,33,40]. The remaining isolates, except one, could be digested with *Xma*I, the neoschizomers of *Sma*I. Neoschizomers have the same recognition sequence but are cut at different positions, providing a solution if one enzyme cannot digest an isolate. This case was also experienced by Chanchaithong et al. [33], who used *Cfr9*I (*Xma*I) as neoschizomers of *Sma*I. Sasaki et al. [4] also used *Xma*I as neoschizomers of *Sma*I. In addition, they used *Asc*I as a restriction enzyme for isolates that both *Sma*I and *Xma*I enzymes could not digest. 

The PFGE analysis revealed extensive genetic diversity among the isolates, with 34 pulsotypes identified by *Sma*I digestion and 14 by *Xma*I digestion, and no typical or predominant PFGE pulsotypes were identified. These *mecA*-positive *S. pseudintermedius* isolates possibly originated from discrete sources, not just from a single source. A similar result of high diversity among isolates was also discovered by previous studies [29,32,33,40,41]. Even though PFGE has high discriminatory power and is considered the gold standard for bacteria typing [42], due to the absence of the standardized PFGE method for *S. pseudintermedius*, the results obtained between laboratories or between studies have been varied and difficult to compare [43]. 

The findings of this study underscore the complexity of *S. pseudintermedius* colonization and infection dynamics in pyoderma-affected dogs. Despite different pulsotypes, the consistent SCC*mec* types and antibiogram patterns among OR-MRSP isolates from individual dogs suggest the horizontal transfer of SCC*mec* elements and other antibiotic-resistant genes within hosts. This highlights the role of SCC*mec* elements in disseminating antibiotic resistance among *S. pseudintermedius* populations, which can complicate treatment strategies. 

In contrast, the variability in phenotypes and genotypes observed among OS-MRSP isolates from a single dog suggests the potential for mixed infections. This finding underscores the importance of comprehensive sampling from multiple sites to characterize resistance profiles and inform effective treatment regimens accurately. The high level of genetic variability, as indicated by the presence of both similar and dissimilar pulsotypes among isolates from the same dog, further complicates the infection dynamics. The detection of identical pulsotypes from different anatomical sites within the same dog indicates that *S. pseudintermedius* can colonize multiple locations, facilitating its persistence and dissemination. 

Our study has limitations due to its sampling strategy, which was confined to diseased dogs. This focus may not fully capture the diversity of the broader canine population. Future research incorporating whole genome analysis could offer a more comprehensive understanding of the genetic relationships among isolates, as well as insights into the antimicrobial resistance mechanisms of both oxacillin-resistant and oxacillin-susceptible *mecA*-positive *S. pseudintermedius*.

## 5. Conclusions

This study underscores the significant challenge posed by *mecA*-positive *S. pseudintermedius* in canine pyoderma, particularly due to the high prevalence of multidrug resistance and the genetic diversity of isolates. The findings highlight the necessity for routine *mecA* screening in diagnostic laboratories to ensure accurate diagnosis and effective treatment. Enhanced surveillance is crucial to managing infections and mitigating the spread of *mecA*-positive strains. Future research should focus on elucidating the mechanisms underlying the oxacillin susceptibility in *mecA*-positive *S. pseudintermedius* isolates and their potential to revert to a resistant phenotype.

## Figures and Tables

**Table 1:** Sample origins and distribution of *Staphylococcus pseudintermedius* isolates positive for the *mecA* gene recovered from 46 enrolled dogs.

Origin of Samples	Number of Dogs (n)		
	One Isolate	Two Isolates ^1^	Three Isolates ^2^
Skin lesions	9	17	0
Nasal cavity	6	0	0
Skin lesions and nasal cavity	0	4	10

^1^ Isolates were obtained from two different sites on the dog. ^2^ Two isolates were from skin lesions, and another isolate was from a nasal swab.

**Table 2:** Characteristics of typeable and non-typeable SCC*mec Staphylococcus pseudintermedius* isolates.

*ccr* Gene Complex	*mec* Gene Complex	SCC*mec* Type	Total Isolates (%)
5 (C)	C2	V	39/87 (45)
1 (A1B1) and 5 (C)	C2	NT	33/87 (38)
1 (A1B1) and 5 (C)	A	NT	2/87 (2)
2 (A2B2) and 5 (C)	B and C2	NT	2/87 (2)
1 (A1B1)	A and C2	NT	1/87 (1)
1 (A1B1) and 5 (C)	Negative	NT	9/87 (10)
5 (C)	A	NT (5A, new combination)	1/87 (1)

NT, non-typeable.

**Table 3:** Genotypic characteristics and multidrug-resistant phenotypes of 33 oxacillin-resistant *mecA*-positive *Staphylococcus pseudintermedius* (OR-MRSP) isolates.

Dog No.	Isolate Code	Origin of Sample	MR/MS Phenotype	MDR Phenotype	PFGE Pulsotype	SCC*mec* Typing		
						*ccr* Gene Complex	*mec* Gene Complex	SCC*mec* Type
4	4 S1	Skin	MR	MDR	*Sma*I-AH	5 (C)	A	NT
	4 S2	Skin	MR	MDR	*Xma*I-D	5 (C), 1 (A1B1)	A	NT
6	6 S1	Skin	MR	MDR	*Sma*I-B	5 (C), 1 (A1B1)	C2	NT
	6 S2	Skin	MR	MDR	*Sma*I-B	5 (C), 1 (A1B1)	C2	NT
	6 N1	Nasal	MR	MDR	*Sma*I-B	5 (C), 1 (A1B1)	C2	NT
11	11 N1	Nasal	MR	MDR	*Xma*I-C	5 (C)	C2	V
12	12 S1	Skin	MR	MDR	*Xma*I-E	5 (C)	C2	V
	12 S2	Skin	MR	MDR	*Xma*I-E	5 (C)	C2	V
	12 N1	Nasal	MR	MDR	nt	5 (C)	C2	V
18	18 S1	Skin	MR	MDR	*Sma*I-K	5 (C)	C2	V
	18 S2	Skin	MR	MDR	*Xma*I-I	5 (C)	C2	V
	18 N1	Nasal	MR	MDR	*Xma*I-I	5 (C)	C2	V
19	19 S1	Skin	MR	MDR	*Sma*I-E	5 (C), 1 (A1B1)	C2	NT
	19 S2	Skin	MR	MDR	*Sma*I-A	5 (C)	C2	V
23	23 S2	Skin	MR	MDR	*Sma*I-AB	5 (C)	C2	V
	23 N1	Nasal	MR	MDR	*Sma*I-AB	5 (C)	C2	V
26	26 S1	Skin	MR	MDR	*Sma*I-S	5 (C)	C2	V
	26 S2	Skin	MR	MDR	*Sma*I-I	5 (C)	C2	V
32	32 S1	Skin	MR	MDR	*Xma*I-G	5 (C), 2 (A2B2)	B, C2	NT
	32S2	Skin	MR	MDR	*Xma*I-G	5 (C), 2 (A2B2)	B, C2	NT
33	33 S1	Skin	MR	MDR	*Sma*I-AE	5 (C)	C2	V
	33 S2	Skin	MR	MDR	*Sma*I-AE	5 (C)	C2	V
36	36 N1	Nasal	MR	MDR	*Sma*I-AB	5 (C), 1 (A1B1)	C2	NT
41	41 S1	Skin	MR	MDR	*Sma*I-AB	5 (C)	C2	V
	41 S2	Skin	MR	MDR	*Sma*I-AB	5 (C)	C2	V
	41 N1	Nasal	MR	MDR	*Sma*I-G	5 (C)	C2	V
42	42 S1	Skin	MR	MDR	*Sma*I-D	5 (C)	C2	V
	42 S2	Skin	MR	MDR	*Xma*I-M	5 (C)	C2	V
	42 N1	Nasal	MR	MDR	*Sma*I-M	5 (C)	C2	V
43	43 S1	Skin	MR	MDR	*Sma*I-AG	5 (C)	C2	V
	43 S2	Skin	MR	MDR	*Sma*I-AC	5 (C)	C2	V
	43 N1	Nasal	MR	MDR	*Sma*I-AC	5 (C)	C2	V
57	57 S2	Skin	MR	MDR	*Sma*I-C	5 (C), 1 (A1B1)	C2	NT

nt, not tested; NT, non-typeable; MR/MS, methicillin-resistant/methicillin-susceptible (based on VITEK results and oxacillin disk diffusion test); MDR, multidrug-resistant (based on VITEK results); Pos, positive; Neg, negative.

**Table 4:** Genotypic characteristics and multidrug-resistant phenotypes of 54 oxacillin-susceptible *mecA*-positive *Staphylococcus pseudintermedius* (OS-MRSP) isolates.

Dog No.	Isolates Code	Origin of Sample	MR/MS Phenotype	MDR Phenotype	PFGE Pulsotype	SCC*mec* Typing		
						*ccr* Gene Complex	*mec* Gene Complex	SCC*mec* Type
7	7 S2	Skin	MS	NO	*Sma*I-Y	5 (C), 1 (A1B1)	C2	NT
8	8 S1	Skin	MS	MDR	*Sma*I-A	5 (C), 1 (A1B1)	C2	NT
	8 S2	Skin	MS	MDR	*Sma*I-A	5 (C), 1 (A1B1)	C2	NT
9	9 S1	Skin	MS	MDR	*Sma*I-A	5 (C), 1 (A1B1)	(-)	NT
	9 S2	Skin	MS	MDR	*Sma*I-R	5 (C), 1 (A1B1)	(-)	NT
	9 N1	Nasal	MS	MDR	*Sma*I-F	5 (C)	C2	V
10	10 S2	Skin	MS	MDR	*Sma*I-P	5 (C), 1 (A1B1)	(-)	NT
13	13 S1	Skin	MS	NO	*Xma*I-L	5 (C), 1 (A1B1)	C2	NT
	13 S2	Skin	MS	NO	*Xma*I-J	5 (C), 1 (A1B1)	A	NT
15	15 S1	Skin	MS	NO	*Sma*I-V	5 (C), 1 (A1B1)	(-)	NT
	15 S2	Skin	MS	NO	*Sma*I-L	5 (C), 1 (A1B1)	A, C2	NT
16	16 S1	Skin	MS	NO	*Sma*I-A	5 (C), 1 (A1B1)	(-)	NT
17	17 S2	Skin	MS	NO	*Sma*I-H	5 (C), 1 (A1B1)	(-)	NT
	17 N1	Nasal	MS	NO	*Sma*I-V	5 (C), 1 (A1B1)	C2	NT
20	20 S1	Skin	MS	NO	*Xma*I-F	5 (C), 1 (A1B1)	C2	NT
	20 S2	Skin	MS	NO	*Sma*I-E	5 (C), 1 (A1B1)	(-)	NT
21	21 N1	Nasal	MS	NO	*Sma*I-W	5 (C)	C2	V
22	22 S1	Skin	MS	NO	*Sma*I-L	5 (C), 1 (A1B1)	C2	NT
25	25 S1	Skin	MS	MDR	*Xma*I-K	5 (C), 1 (A1B1)	(-)	NT
	25 S2	Skin	MS	MDR	*Xma*I-K	5 (C), 1 (A1B1)	C2	NT
27	27 S1	Skin	MS	NO	*Sma*I-N	5 (C), 1 (A1B1)	C2	NT
	27 S2	Skin	MS	NO	*Sma*I-N	5 (C), 1 (A1B1)	(-)	NT
28	28 S1	Skin	MS	NO	*Sma*I-O	5 (C), 1 (A1B1)	C2	NT
29	29 S2	Skin	MS	NO	*Sma*I-G	5 (C), 1 (A1B1)	C2	NT
30	30 S1	Skin	MS	NO	*Sma*I-AE	5 (C)	C2	V
	30 S2	Skin	MS	NO	*Sma*I-AE	5 (C)	C2	V
31	31 S2	Skin	MS	NO	*Xma*I-N	5 (C)	C2	V
	31 N1	Nasal	MS	NO	*Xma*I-N	5 (C)	C2	V
34	34 S1	Skin	MS	MDR	*Sma*I-U	5 (C), 1 (A1B1)	C2	NT
	34 S2	Skin	MS	MDR	*Sma*I-U	5 (C), 1 (A1B1)	C2	NT
35	35 S2	Skin	MS	MDR	*Xma*I-H	5 (C)	C2	V
37	37 N1	Nasal	MS	NO	*Sma*I-Q	5 (C)	C2	V
44	44 S1	Skin	MS	NO	*Sma*I-Z	5 (C)	C2	V
	44 S2	Skin	MS	NO	*Sma*I-AA	5 (C), 1 (A1B1)	C2	NT
	44 N1	Nasal	MS	NO	*Sma*I-V	5 (C)	C2	V
45	45 N1	Nasal	MS	NO	*Sma*I-AF	5 (C), 1 (A1B1)	C2	NT
46	46 S1	Skin	MS	MDR	*Sma*I-E	5 (C), 1 (A1B1)	C2	NT
	46 S2	Skin	MS	MDR	*Sma*I-E	5 (C), 1 (A1B1)	C2	NT
48	48 S1	Skin	MS	MDR	*Sma*I-V	5 (C), 1 (A1B1)	C2	NT
	48 S2	Skin	MS	NO	*Sma*I-C	5 (C), 1 (A1B1)	C2	NT
49	49 S1	Skin	MS	MDR	*Xma*I-A	5 (C), 1 (A1B1)	C2	NT
	49 S2	Skin	MS	MDR	*Xma*I-A	5 (C), 1 (A1B1)	C2	NT
56	56 S1	Skin	MS	NO	*Sma*I-V	5 (C)	C2	V
	56 S2	Skin	MS	NO	*Sma*I-AD	5 (C)	C2	V
	56 N1	Nasal	MS	MDR	*Sma*I-T	5 (C), 1 (A1B1)	C2	NT
58	58 S2	Skin	MS	MDR	*Xma*I-B	5 (C), 1 (A1B1)	C2	NT
	58 N1	Nasal	MS	MDR	*Sma*I-J	5 (C), 1 (A1B1)	C2	NT
59	59 S2	Skin	MS	MDR	*Xma*I-B	5 (C), 1 (A1B1)	C2	NT
60	60 S1	Skin	MS	MDR	*Sma*I-R	5 (C)	C2	V
	60 S2	Skin	MS	NO	*Sma*I-X	5 (C), 1 (A1B1)	C2	NT
	60 N1	Nasal	MS	NO	*Sma*I-X	5 (C), 1 (A1B1)	C2	NT
62	62 N1	Nasal	MS	MDR	*Sma*I-A	5 (C)	C2	V
63	63 S1	Skin	MS	MDR	*Sma*I-R	5 (C)	C2	V
	63 S2	Skin	MS	MDR	*Sma*I-R	5 (C)	C2	V

NT, non-typeable; MR/MS, methicillin-resistant/methicillin-susceptible (based on VITEK results and oxacillin disk diffusion test); MDR, multidrug-resistant (based on VITEK results).

## Data Availability

The data presented in this study are available on request from the corresponding author.

## Supplementary Materials

The following supporting information can be downloaded at https://www.mdpi.com/article/10.3390/ani14172613/s1; Figure S1: *Sma*I-pulsed-field gel electrophoresis (PFGE) dendrogram of 65 *mecA*-positive *Staphylococcus pseudintermedius* isolates; Figure S2: *Xma*I-pulsed-field gel electrophoresis (PFGE) dendrogram of 21 *mecA*-positive *Staphylococcus pseudintermedius* isolates.
