# Supplementary material for: Characterization of Oxacillin-Resistant and Oxacillin-Susceptible mecA-Positive Staphylococcus pseudintermedius from Skin Lesions and Nasal Cavities of Dogs with Clinical Pyoderma
Source: Animals (Basel). 2024 Sep 8;14(17):2613. doi: 10.3390/ani14172613 (PMC11394641; doi:10.3390/ani14172613)

Supplementary Figure S1. *Sma*I-pulsed-field gel electrophoresis (PFGE) dendrogram of 65 *mecA* positive *Staphylococcus pseudintermedius* isolates

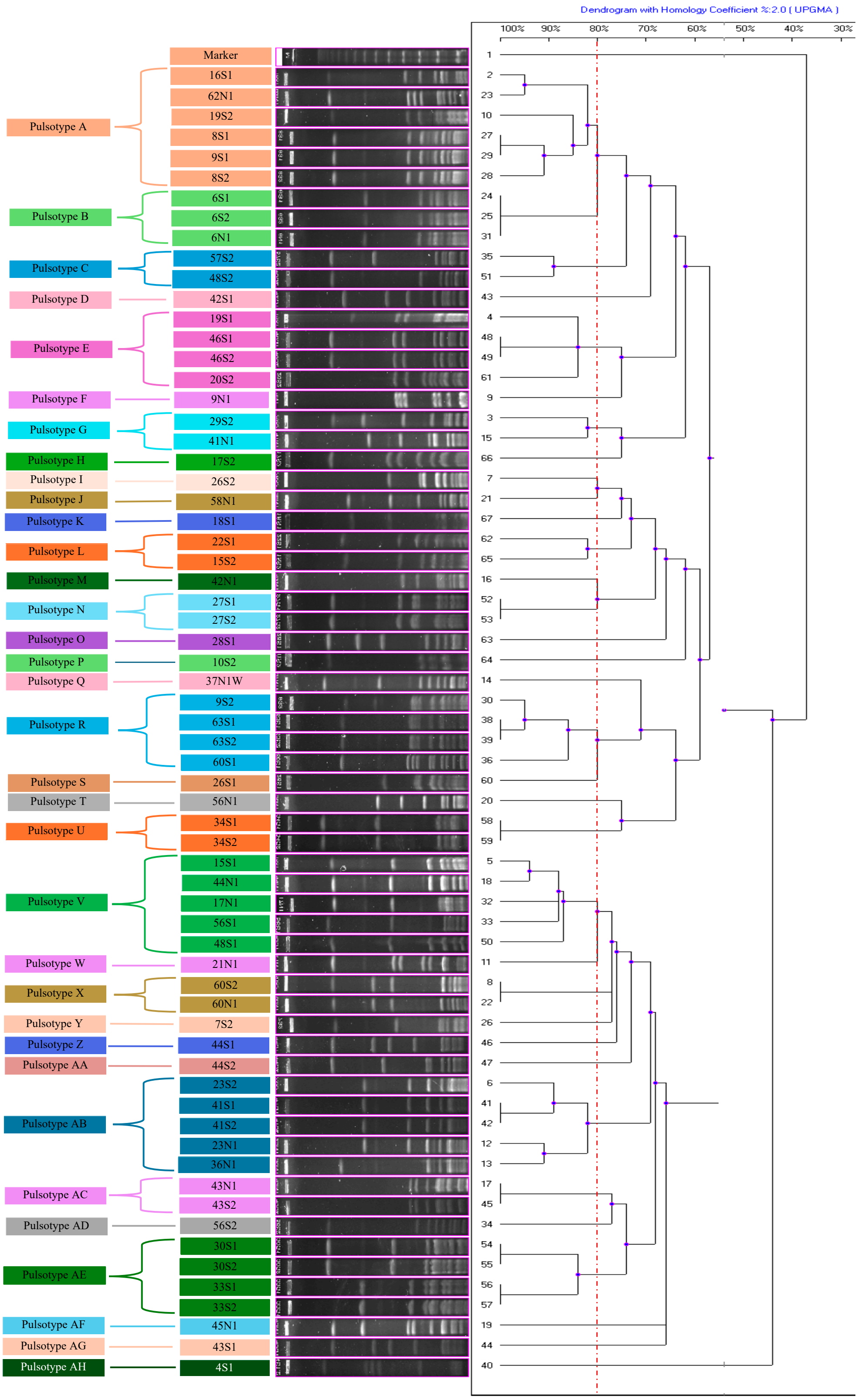

Supplementary Figure S2. *Xma*I-pulsed-field gel electrophoresis (PFGE) dendrogram of 21 *mecA* positive *Staphylococcus pseudintermedius* isolates

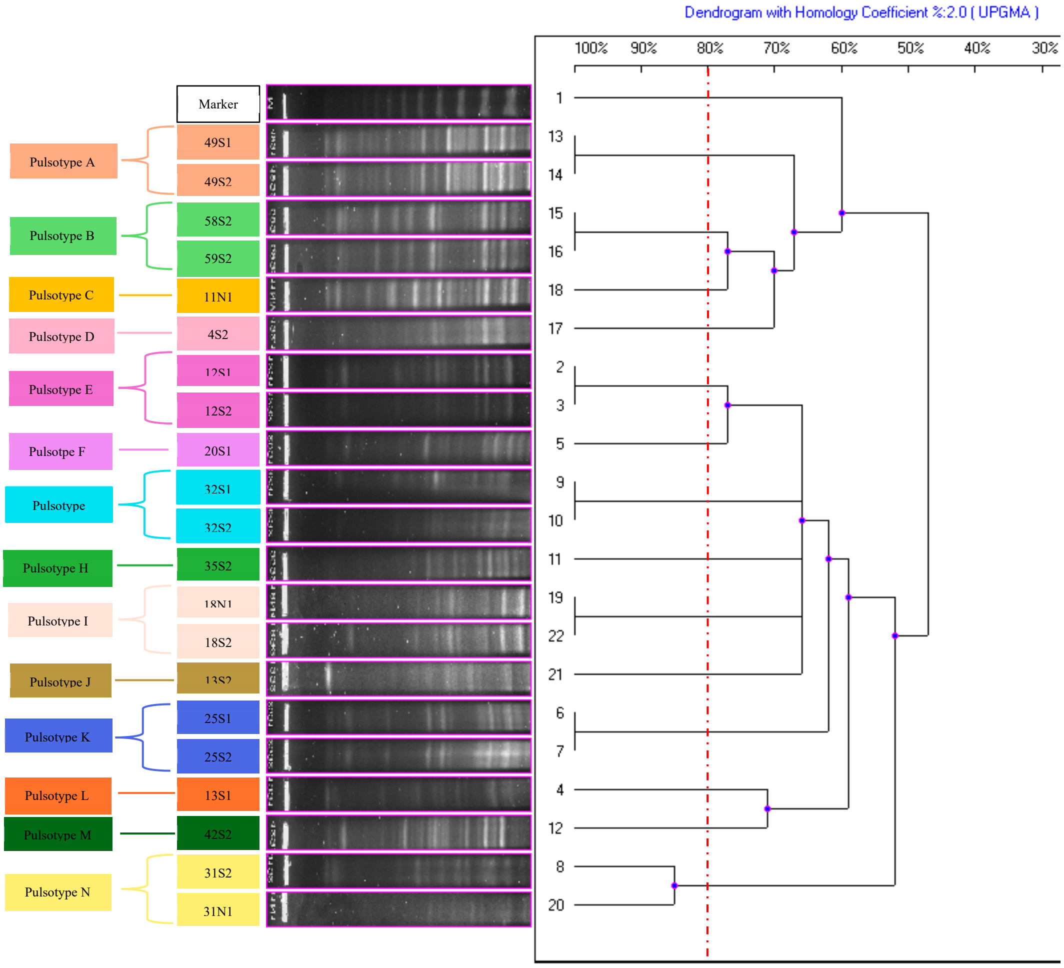

Supplement: Supplementary file 1 [file animals-14-02613-s001.zip › Supplementary Figures.pdf]
